# Characterisation of Crevice and Pit Solution Chemistries Using Capillary Electrophoresis with Contactless Conductivity Detector

**DOI:** 10.3390/ma6104345

**Published:** 2013-09-30

**Authors:** Mengyan Nie, Julian A. Wharton, Andy Cranny, Nick R. Harris, Robert J.K. Wood, Keith R. Stokes

**Affiliations:** 1 National Centre for Advanced Tribology at Southampton (nCATS), Faculty of Engineering and the Environment, University of Southampton, Southampton SO17 1BJ, UK; E-Mails: j.a.wharton@soton.ac.uk (J.A.W.); r.wood@soton.ac.uk (R.J.K.W.); krstokes@mail.dstl.gov.uk (K.R.S.); 2 Electronics and Computer Science, Faculty of Physical Sciences and Engineering, University of Southampton, Southampton SO17 1BJ, UK; E-Mails: awc@ecs.soton.ac.uk (A.C.); nrh@ecs.soton.ac.uk (N.R.H.); 3 Physical Sciences Department, Defence Science and Technology Laboratory (Dstl), Porton Down, Salisbury SP4 0JQ, UK

**Keywords:** structural health monitoring, corrosion solution chemistries, corrosion monitoring, capillary electrophoresis, contactless conductivity detection

## Abstract

The ability to predict structural degradation in-service is often limited by a lack of understanding of the evolving chemical species occurring within a range of different microenvironments associated with corrosion sites. Capillary electrophoresis (CE) is capable of analysing nanolitre solution volumes with widely disparate concentrations of ionic species, thereby producing accurate and reliable results for the analysis of the chemical compositions found within microenvironment corrosion solutions, such as those found at crevice and pit corrosion sites. In this study, CE with contactless conductivity detection (CCD) has been used to characterize pitting and crevice corrosion solution chemistries for the first time. By using the capillary electrophoresis with contactless conductivity detection (CE-CCD) system, direct and simultaneous detection of seven metal cations (Cu^2+^, Ni^2+^, Fe^3+^, Fe^2+^, Cr^3+^, Mn^2+^, and Al^3+^) and chloride anions was achieved with a buffer solution of 10 mM 2,6-pyridinedicarboxylic acid and 0.5 mM cetyltrimethylammonium hydroxide at pH 4 using a pre-column complexation method. The detection limits obtained for the metal cations and chloride anions were 100 and 10 ppb, respectively. The CE-CCD methodology has been demonstrated to be a versatile technique capable of speciation and quantifying the ionic species generated within artificial pit (a pencil electrode) and crevice corrosion geometries for carbon steels and nickel-aluminium bronze, thus allowing the evolution of the solution chemistry to be assessed with time and the identification of the key corrosion analyte targets for structural health monitoring.

## 1. Introduction

The failure of a structure, such as an airframe or marine vessel, can cause considerable economic loss, and even loss of life. Using an array of sensors to continuously monitor such structures, structural health monitoring can provide an early indication of problems such as damage to the structure from fatigue, corrosion or impact, and this information can be used to undertake corrective action before the damage develops to a stage where a catastrophic failure occurs [1,2]. However, there are a wide variety of corrosion types that routinely occur in aircraft and marine structures. For example, corrosion can occur in situations where the metal components are generally expected to be corrosion-resistant and can result in the destruction of integrity of metal-metal and metal-nonmetal junctions (crevice corrosion) such as may exist at bolted and lock-tight joints, flanges, and poor quality welds. For ferrous metals and aluminium alloys, the chemical microenvironment in these affected areas becomes acidic and eventually the crevice becomes sufficiently aggressive to depassivate the metallic surface [3]. Likewise, crevice corrosion can also occur for copper-based systems, for example nickel-aluminium bronze (NAB) where there is a selective phase attack within the occluded zone plus an additional accelerated attack at the crevice edge [4].

Although non-destructive evaluation for corrosion detection is becoming available, corrosion is often found using visual inspection methods. This means that corrosion of internal or inaccessible structures could go undetected [5]. To date, the only practical solution to check for corrosion damage has been to strip and inspect. Frequently, no corrosion is found, but the time and cost of performing the inspection has already been lost and damage may even result if the refurbishment is to a lower standard than the original construction. The objective of corrosion surveillance is to predict attack before significant damage is sustained, the condition of components is thus monitored whilst in service and not just intermittently at routine inspections. This minimises the inspection requirements yet ensures that maintenance is carried out as it becomes necessary. Recognition of the advantage gained from predictive management of corrosion is becoming more widespread, following such developments in the nuclear, chemical and offshore oil industry [6,7], with cost savings achieved equivalent to approximately half the cost of using the conventional maintenance approach [8].

However, the ability to predict structural degradation in-service is limited in many cases by a lack of understanding and detailed knowledge of the evolving chemical processes occurring within a range of different microenvironments containing micro-solution volumes. Generally, there is a lack of data with regard to the levels of metal cations within the microenvironments that form in situations such as crevice corrosion due to the small quantities of materials of interests and the inherent analytical difficulty of measuring small amounts of one species in the presence of large amounts of another. CE has been proven to be a powerful and robust analytical technique for corrosion solution analysis with a low level (e.g., parts per million, ppm) detection of some metallic cations and inorganic anions in the presence of high levels (in the order of 1 M) of chloride ions [9,10,11,12,13,14].

Capillary electrophoresis (CE) separates different ionic species within a buffer solution filled capillary under the influence of an external electric field based on differences in their electrophoretic mobilities. Only requiring very small sample volumes of less than 10 µL to detect both anions and cations, it offers high resolution, fast analysis, and simple sample preparation methodology. The most widely used mode of detection in CE is ultraviolet (UV) absorbance detection, which works well for organic species with chromophoric moieties. Unfortunately, due to their lack of significant UV absorbance, most inorganic anions and metal cations can only be detected by indirect UV detection, a method which is relatively insensitive and has a limited linear range. For example, Kelly *et al.* [11,12,13,14] have quantified local chemistries within the corrosion-induced blisters in organic coatings on aluminium alloys using CE with UV detection, but four different buffer electrolytes were used for the indirect UV detection of inorganic anions and cations existing within the corrosion microenvironments. However, these small inorganic ions can be directly determined with simpler buffer compositions by measuring conductivity changes in the capillary with contactless conductivity detection (CCD). Capillary electrophoresis with contactless conductivity detection (CE-CCD) has been proven to allow direct and simultaneous detection of inorganic anions and cations at extremely low concentration levels down to parts per billion (ppb) and even parts per trillion ranges [15,16,17]. In addition to conventional CE, CCD has also proven to be preferred and well suited for micro-CE, due to its high sensitivity, low cost, simple fabrication and easy implementation [17,18,19].

In this paper, a capillary electrophoresis analysis approach using contactless conductivity detection has been established for the first time to allow direct and simultaneous detection of chloride anions and the most common metal alloy cations, including cupric ions, nickel ions, ferric ions, ferrous ions, chromium ions, manganese ions and aluminium ions. Using this new CE methodology, the solution chemistries sampled from either a pencil electrode (an artificial pit electrode, often called one-dimensional pit geometry) or crevice corrosion assembly for carbon steel and nickel-aluminium bronze have been determined.

## 2. Experimental Section

### 2.1. Chemicals and Test Solutions

All reagents (analytical grade) were supplied by Sigma-Aldrich (Poole, UK) and dissolved in 18 MΩ cm deionised water as received. All the standard solutions of 2000 ppm copper ions, nickel ions, ferric ions, ferrous ions, chromium ions, manganese ions and aluminium ions were prepared from their chloride salts. The CE buffer solution was prepared using a solution having appropriate amounts of 2,6-pyridinedicarboxylic acid (PDCA) with additional appropriate amounts of cationic surfactant cetyltrimethylammonium hydroxide (CTAH) or tetramethylammonium hydroxide (TMAH), and the pH was adjusted with 2 M sodium hydroxide.

### 2.2. Specimen Materials

**Figure 1:**
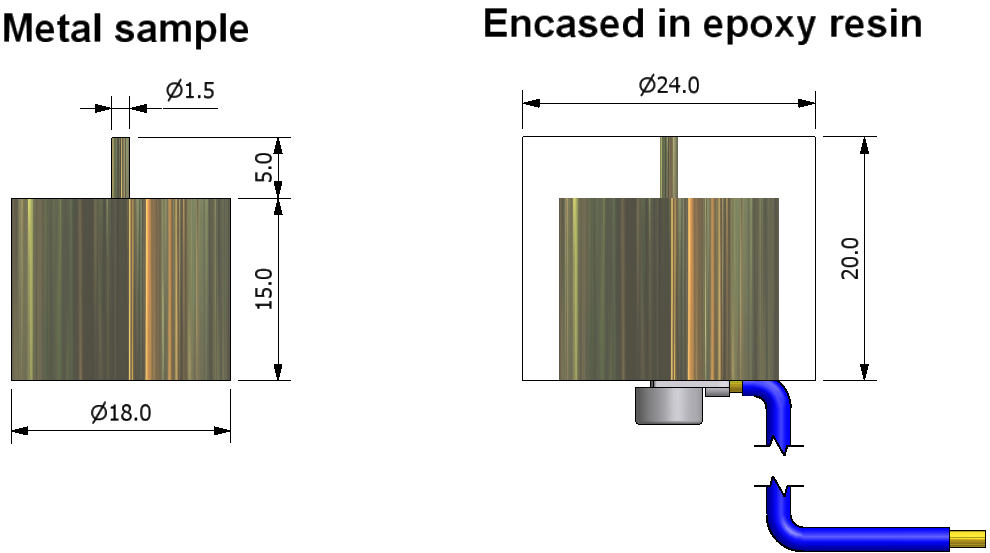
Pencil electrode assembly for accelerated corrosion experiments (all dimensions in mm).

A carbon steel, EN3A grade (equivalent to 090M20–BS970 Part 1, AISI 1020 or UNS G10200), was used for accelerated corrosion tests (chemical composition: Fe balance, C 0.17–0.23, Mn 0.60–0.90, S 0.05 (max.) wt %). Cast nickel-aluminium bronze (UNS C95800, British Naval spec. NES 747 Part 2) was also used for accelerated corrosion tests (chemical composition: Cu balance, Al 9.32, Ni 5.38, Fe 5.00, Mn 1.10, Si 0.05 wt %). The specimens for accelerated corrosion tests were machined as pencil electrodes and embedded in epoxy resin, as shown in Figure 1. Prior to testing, the pencil electrodes were wet abraded sequentially with 600 and 1200 grit SiC papers and polished using 6 µm and 1 µm diamond pastes, then degreased in acetone and air-dried. In addition, a high strength low alloy Q1N carbon steel (HY80) was used for the Cortest crevice corrosion tests (chemical composition: Fe balance, Ni 2.25–3.25, Cr 1.00–1.80, Mo 0.3–0.6, Si 015–0.35, C 0.18 (max.) wt %). The Q1N specimens for Cortest experiments were 50 mm × 50 mm × 5 mm with a central 12 mm diameter hole. Prior to testing, the Cortest specimens were wet abraded with 600 SiC grit paper on both sides to provide a uniform surface finish, then degreased in acetone and air-dried.

### 2.3. Instrumentation

The CE experiments were performed using a Prince Technologies PrinCE-560 capillary electrophoresis system (Prince Technologies B.V., Emmen, The Netherlands) with a TraceDec contactless conductivity detector. All CE measurements and data processing were carried out with Data Acquisition and Analysis Software (DAx 8 Analysis, Prince Technologies B.V., Emmen, The Netherlands). A bare fused silica capillary was used for all the CE measurements with an inner diameter of 50 μm, total length of 90 cm and effective length of 60 cm. The CE separation was carried out at a temperature of 20 °C.

Electrochemical tests were performed within a Faraday cage using a Gamry Reference 600 potentiostat with Gamry Framework and Software Package 5.63 (Gamry Instruments, Warminster, PA, USA). A standard reference electrode of Ag/AgCl in 3.5 M KCl was used for all the electrochemical measurements, and a graphite rod was used as the counter electrode. pH values were measured using a Hanna pH meter HI211 (Hanna Instruments, RI, USA) with a MI-4156 combination microelectrode, with an outer diameter of 1.3 mm (Microelectrodes, Inc., Bedford, NH, USA). All electrochemical testing was carried out in 3.5% NaCl solution at room temperature.

### 2.4. Pencil Electrode Assembly (Artificial One-Dimensional Pit)

Accelerated corrosion processes were investigated for the EN3A carbon steel and NAB to identify those ionic species that are indicative of corrosion occurring. All the accelerated corrosion tests were performed with pencil electrode assemblies as shown in Figure 1. When encased in epoxy resin, immersed in a 3.5% NaCl solution and appropriately biased using a potentiostat, the small surface area of the exposed pencil electrode is progressively corroded leaving a cavity within the epoxy resin containing a small volume of the corrosion products, which were collected with a micropipette at predefined time intervals and diluted to 4 to 200 folds with the running buffer solution for pre-column complexation treatment and CE analysis.

### 2.5. Cortest Crevice Corrosion Assemblies

**Figure 2:**
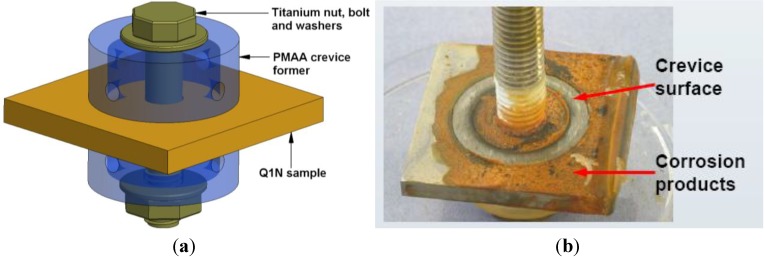
Schematic of (**a**) a Cortest crevice corrosion assembly; and (**b**) crevice corrosion sample of Q1N after immersion in 3.5% NaCl solution for 24 days.

As described in [9], crevice formers were created using the Cortest assembly (large single crevice area of 4.6 cm^2^) manufactured from polymethylmethacrylate (PMMA) and fastened using commercially pure titanium nuts, bolts and washers, as shown in Figure 2. Crevice assembly tightness was controlled using a torque wrench set to 0.6 N.m. To prevent electrical connection between the Q1N specimen and the titanium parts, ploytetrafluoroethylene (PTFE) tape was used to electrically insulate the bolt. In total eight crevice corrosion assemblies were immersed into a tank containing 4.6 L of a continuously aerated 3.5 wt % NaCl test solution (bulk solution). The bulk solution dissolved oxygen content was measured with a Hanna Instruments HI9143 probe and found to be approximately 7.0 ppm.

At set time intervals, a single crevice assembly was retrieved from the bulk solution and placed in a freezer for about half an hour. This step was taken to ensure that crevice solution samples remained on the specimen surface during the crevice former disassembly. The melted solution was collected from the crevice surface in the region immediately under the crevice former using a micropipette. Prior to CE analysis, the collected samples were mixed with the CE running buffer solution for pre-column complexation and 4 to 200-folds dilution.

## 3. Results and Discussion

### 3.1. Capillary Electrophoresis Method for Corrosion Solution Chemistries Analysis

The most common alloying components are copper, nickel, iron, chromium, manganese and aluminium. Most of the widely used marine and aerospace alloys normally contain four to six of these metal alloying elements. In order to identify the possible metal cations produced during corrosion processes, it is important to conduct an analysis such that it is capable of detecting all of the corrosion products in a single CE run, especially when considering the very small corrosion solution volumes that can occur. To date, the separation of all aforementioned seven metal cations as well as chloride ions in a single analysis run has previously not been reported for CE or ion chromatography.

Anions and cations migrate in opposite directions in CE, and it is difficult to simultaneously determine metal cations and chloride anions in a single CE run. Thus, all the metal ions need to be transformed at first into negatively charged complexes to achieve simultaneous separation of all the target metal cations with negatively charged chloride ion in a single CE run. In addition, because these metal ions possess similar mobilities, it is also necessary to use a chelating agent to enhance separation efficiency and selectivity of these metal ions in order to realize the simultaneous and direct determination of these target metal ions in a single CE run. It has been reported that PDCA can form highly stable, negatively charged complexes with some heavy metal ions [9,20,21,22,23]. PDCA was therefore used as a complexation agent and running buffer additive to establish CE methodology in our experiments. A buffer solution pH range of 3.5 to 4.0 was also reported as having achieved best separation selectivity and sensitivity for metal ions [9,20,21,22,23]. In this study, a number of parameters, including PDCA concentrations in the buffer solution, surfactant types and their concentrations in the buffer solution, pH, sample pre-treatment methods, separation temperature and applied potentials, were investigated to optimise the separation conditions. The results from our investigations into the effect of buffer solution composition on metal ion separation is summarised in Table 1, along with the effects of employing on-column or pre-column complexation sample pre-treatment methodologies.

For these optimisation experiments, all the measurements were performed with a bare fused silica capillary at a temperature of 20 °C with a running time of 1 h by using the standard metal ion solutions prepared from chloride salts of metal ions with a concentration of 10 ppm. For all the measurements, the peak for chloride ion was always detected and completely separated from those for the metal ions.

Table 1 shows that regardless of the buffer solution composition, better separation was achieved with pre-column complexation than on-column complexation. The results also show that when no surfactant was included in the buffer solution, the baseline signal at the detector exhibited significant levels of noise, making it difficult to discriminate peaks in the conductivity signal attributable to the different ions (*i.e.*, selectivity and sensitivity were poor). It was also observed that when CTAH was employed as the surfactant, a lower concentration of PDCA produced better separation efficiency irrespective of the complexation method used. Further experiments with the buffer solution containing 5 mM PDCA and 0.25 mM CTAH confirmed that the optimum buffer solution for reliable separation of all of the ions of interest is 10 mM PDCA and 0.5 mM CTAH at pH 4.0.

**Table 1:** Optimisation of buffer solution and complexation method for separation of seven metal ions.

PDCA	Surfactant	Complexation	Separation results
20 mM	No surfactant	On-column	Very noisy signal baseline. No peaks observed for chloride or metal ions.
20 mM	No surfactant	Pre-column	Very noisy baseline.Cu^2+^ and Ni^2+^ not separated and exhibit same migration time.
20 mM	0.5 mM CTAH	On-column	No separation detected for metal ions.
20 mM	0.5 mM CTAH	Pre-column	No peak detected for Cu^2+^, Ni^2+^ and Mn^2+^ ions. One peak observed for Al^3+^ and another peak for Fe^2+/3+^ and Cr^3+^ ions.
10 mM	5 mM TMAH	On-column	Cu^2+^, Ni^2+^ and Fe^2+^ not separated. Mn^2+^ completely separated from other metal ions. Al^3+^, Cr^3+^ and Fe^3+^ ions not detected.
10 mM	5 mM TMAH	Pre-column	Cu^2+^ and Ni^2+^ partially separated. Mn^2+^ completely separated from other metal ions. Remaining metal ions not separated.
10 mM	0.5 mM CTAH	On-column	Cu^2+^ and Ni^2+^ ions completely separated from each other. Remaining metal ions not detected.
10 mM	0.5 mM CTAH	Pre-column	Best separation with all 7 metal ions separated into 7 peaks.

The electric field strength within the capillary is another important parameter in CE optimisation since it directly governs electrophoretic migration. The electric field strength is changed when either the applied voltage is varied or the capillary length is altered. In CE, increasing the voltage could improve separation resolution and shorten the analysis time, but the production of heat possibly limits the resolution improvement. Therefore, separation of all the target ions was investigated under different field strengths with applied voltages of −10 kV, −15 kV and −30 kV. The obtained results indicate that reasonable separation resolution and analysis time was achieved under an applied voltage of −15 kV with the used fused silica capillary under the optimized buffer solution compositions.

**Figure 3:**
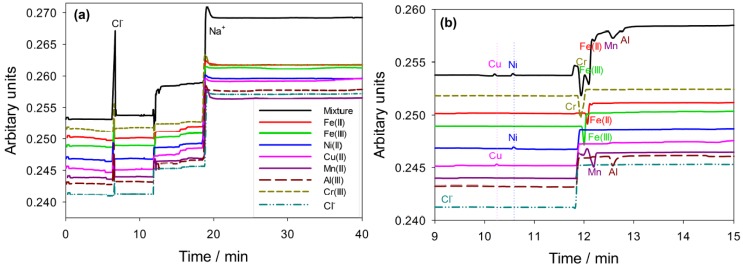
Electropherograms showing separation of seven individual metal ions and chloride ion at the 10 ppm level and when in a mixture, under the optimised conditions described in the text.

As a result, the best separation for all the seven metal cations and chloride anion can be achieved with the buffer solution of 10 mM PDCA and 0.5 mM CTAH at pH 4 using a pre-column complexation method and with the analysis performed at a temperature of 20 °C with an applied voltage of −15 kV. Electropherograms demonstrating the ion separation efficiency under these conditions are displayed in Figure 3. Under the optimized conditions, chloride ion appeared with a very sharp peak before the seven metal ions due to its high mobility, see Figure 3a. At the concentration of 10 ppm, all seven metal ions were separated into individual peaks. Moreover, as demonstrated in Figure 3b, the peaks for Cu^2+^ and Ni^2+^ ions are positive while those for Fe^2+^, Fe^3+^, Mn^2+^, Cr^3+^, and Al^3+^ ions are negative. This indicated that compared to the conductivity of the running buffer solution, the conductivities within the zones formed by the complexation of Cu^2+^ ions with PDCA and the complexation of Ni^2+^ ions with PDCA during the CE separation are greater, while those for PDCA and the other five metal ions are lower.

### 3.2. Calibration Curves for CE-CCD Analysis of Chloride and Metallic Ions

**Figure 4:**
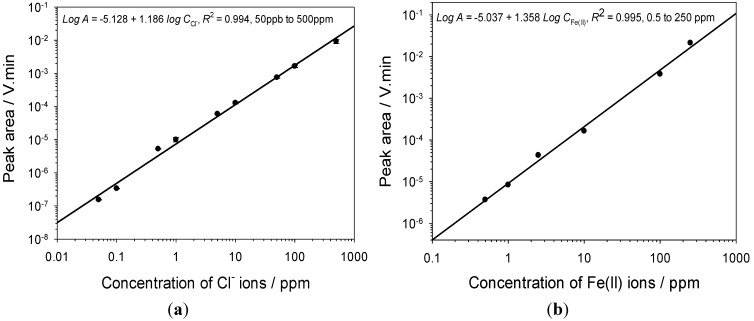
(**a**) Calibration curves for chloride ions; and (**b**) ferrous ions obtained under optimised conditions.

For the CE analysis, sample component identification is achieved by comparing the migration times with those of standard solutions obtained under the same experimental conditions. In order to quantify the composition of the corrosion sample components, calibration curves for each metal ion were first established by plotting the sample concentration *versus* peak area. Under the optimised conditions, CE measurements were carried out for chloride, ferrous, ferric, manganese, chromium, aluminium, cupric and nickel ions in the concentration range 10 ppb to 500 ppm. For all ions investigated, linear relationships were observed with all the coefficients of determination *R*^2^ above 0.99 between the logarithm of the peak area and the logarithm of the concentration over the range 2.5 ppm to 250 ppm. For chloride ions, the linear relationship extended down to the 50 ppb level and the limit of detection for chloride ion was determined as 10 ppb, as shown in Figure 4.

### 3.3. Solution Chemistries Analysis of Accelerated Pitting Corrosion of EN3A Carbon Steel

**Figure 5:**
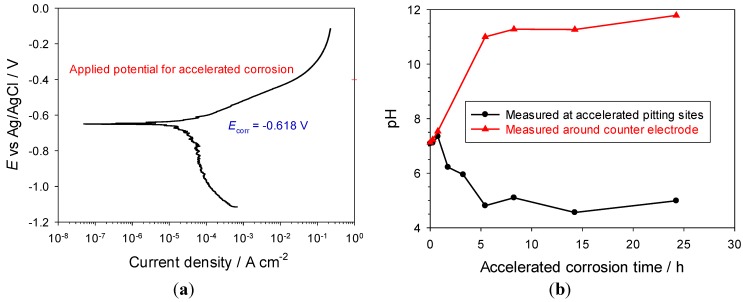
(**a**) Polarisation behaviour at scan rate of 0.150 mV·s^−1^; and (**b**) pH changes at accelerated corrosion sites as well as around counter electrode with accelerated corrosion time at applied potential of −0.400 V for the EN3A carbon steel pencil electrode in 3.5% NaCl solution.

Potentiodynamic polarisations were performed for the carbon steel pencil electrode assembly in a 3.5% NaCl solution to determine an appropriate potential for the accelerated corrosion study. Figure 5 shows the EN3A carbon steel exhibited typical polarisation behaviour for an actively corroding metal, with the corrosion potential (*E*_corr_) at −0.618 V. An applied potential of −0.400 V, where carbon steel is within an active corrosion region, was selected for the accelerated corrosion tests in order to rapidly generate corrosion products within a microenvironment for CE identification.

The pH change was also monitored at the corrosion site of the pencil working electrode and around the counter electrode at pre-determined time intervals. As shown in Figure 5, the pH of the solution around the counter electrode initially increased and reached a value of pH 11.5 after 5 h. By comparison, the solution pH initially decreased at the corrosion site and then stabilised at around pH 5.5 after 5 h.

The predominant cathodic process in an aerated 3.5% NaCl solution is oxygen reduction reaction (ORR):

O_2_ + H_2_O + 4e^−^ → 4OH^−^ (1)

In these circumstances, the products of the ORR are hydroxide ions, thus the solution around the counter electrode (cathode) becomes alkaline during the accelerated corrosion test.

The dissolution of the EN3A pencil electrode resulted in the formation of a pit cavity within the epoxy resin. Ferrous ions are initially considered to form as a primary corrosion product:

Fe → Fe^2+^ + 2e^−^ (2)

As corrosion continues this will lead to an increase in the concentration of dissolved metal ions within the pit cavity, and oxides or hydroxides are precipitated [24]. These hydrolysis and precipitation reactions cause acidification within the pit cavity, in accordance with the following reactions.


Fe^2+^ + H_2_O → Fe(OH)^+^ + H^+^ (3)

Fe(OH)^+^ + H_2_O → Fe(OH)_2_ + H^+^ (4)

2Fe^2+^ + 3H_2_O → Fe_2_O_3_ + 6H^+^ + 2e^−^ (5)

3Fe^2+^ + 4H_2_O → Fe_3_O_4_ + 8H^+^ + 2e^−^ (6)

**Figure 6:**
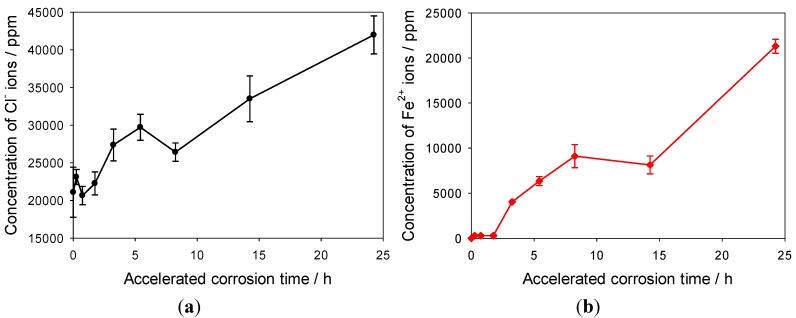
Concentration profiles of (**a**) chloride; and (**b**) ferrous ions as a function of accelerated corrosion time for the freshly polished carbon steel pencil electrode.

In the chloride background media, the increase in the concentrations of Fe^2+^ and H^+^ cations in the cavity will be counterbalanced by an increase in the concentration of Cl^−^ ions, driven by diffusion from the bulk solution towards the cavity interior to maintain electroneutrality. This is confirmed by the chemical analysis results of corrosion solutions collected from the pit cavity during the accelerated corrosion process, see Figure 6. From the CE analysis, the concentration of chloride and ferrous ions reach about 42,000 ppm (1.2 M in terms of NaCl) and 21,000 ppm (0.4 M in terms of FeCl_2_) after 24 h, respectively.

### 3.4. Solution Chemistries Analysis of Cortest Crevice Corrosion of Q1N Carbon Steel

**Figure 7:**
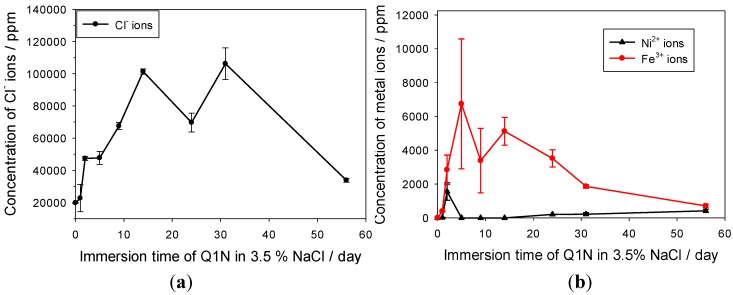
Concentration profiles of (**a**) chloride; (**b**) ferric and nickel ions as a function of immersion time for Q1N in 3.5% NaCl solution.

Q1N is a low alloy carbon steel and it exhibited a similar polarisation behaviour as that observed for the EN3A carbon steel. However, due to nickel and chromium contents, the corrosion potential at −0.570 V was slightly more positive than −0.618 V of EN3A steel in 3.5% NaCl solution. The CE analysis revealed the presence of ferric ions and nickel ions, as well as chloride within the Q1N crevice solution. The concentration profiles of the chloride and metal ions with time were established for the Q1N crevice corrosion for a period of about 8 weeks, see Figure 7. As expected, in order to maintain charge neutrality, the chloride ions were drawn into the crevice site, and the concentration of chloride ions in the crevice increased significantly with corrosion time. The chloride ion concentration in the crevice solution reached a maximum of 106,000 ppm (*ca.* 3.0 M in terms of NaCl) after 2 weeks, before decreasing to 40,000 ppm after 8 weeks. The Fe^3+^ and Ni^2+^ ions exhibited a similar concentration profile with time, rapidly increasing during the initial corrosion phase and then gradually decreasing. The observed trends in concentration profiles with time indicated that crevice corrosion of Q1N activated immediately once immersed in NaCl solution, followed by a resilient period, possibly due to precipitation of metal hydroxides and oxides on the metallic surface (thus hindering corrosion progress). The ferric ions detected within the crevice corrosion solution reached a concentration maximum of 6,700 ppm (*ca.* 0.12 M in term of FeCl_3_) at day 5, while the highest level of nickel ions (1,500 ppm, 0.026 M in terms of NiCl_2_) was detected at the initial stage of crevice corrosion, just after 2 day in the 3.5% NaCl test solution.

### 3.5. Solution Chemistries Analysis of Accelerated Pitting Corrosion of NAB

NAB is a complex copper-based alloy and it is utilised in numerous marine applications due to its good corrosion performance. As previously reported [25], the microstructure of NAB is predominantly constituted of copper-rich α-phase and retained martensitic β-phase, surrounded by a series of intermetallic κ-phases all different in shape and composition. In comparison with the two carbon steels, NAB exhibits a more localised corrosion mechanism. NAB corrosion usually initiates at selective sites, associated with the α-phase where dissolution of copper components occurs to form a cuprous dichloride anion complex (CuCl_2_^−^) in the presence of concentrated chloride, or they precipitate on the metal surface in the form of cuprous chloride (CuCl, a white solid). In neutral solutions, the presence of high concentrations of CuCl_2_^−^ at the metal surface may result in a hydrolysis reaction and the formation of Cu_2_O according to:

2CuCl_2_^−^ + H_2_O → Cu_2_O + 2H^+^ +4Cl^−^ (7)

Alternatively, CuCl precipitates in a chloride media may lead to further Cu_2_O growth:

2CuCl + H_2_O → Cu_2_O + 2H^+^ + 2Cl^−^ (8)

**Figure 8:**
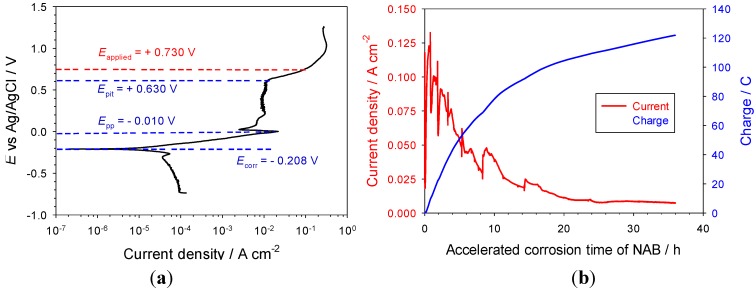
(**a**) Polarisation behaviour at scan rate of 0.150 mV·s^−1^; and (**b**) electrochemical response with accelerated corrosion time at applied potential of +0.730 V for NAB pencil electrode in 3.5% NaCl solution.

As shown in Figure 8, NAB exhibits active-passive corrosion behaviour in the 3.5% NaCl test solution. In the active region between the corrosion potential (*E*_corr_ = −0.208 V) and the pseudo-passivation potential (*E*_pp_ = −0.010 V), the predominant process is the dissolution of copper to form CuCl_2_^−^, or the formation of CuCl on the NAB surface, both of which further hydrolyse to form a protective cuprous oxide layer [see Equations (7) and (8)] [9,25]. Due to the porous microstructure and the electrical conductivity of the Cu_2_O passive layer, the current density is maintained at a relatively high and steady level in the passive region, where the corrosion rates are about two orders of magnitude higher than the corrosion rate at the corrosion potential. Once breakdown of the oxide protective film starts at *E*_pit_ = +0.630 V, the current increased significantly with applied potential. Therefore, for these accelerated corrosion tests, an applied potential of +0.730 V was chosen, which is 0.1 V greater than the passive film breakdown potential, so that corrosion products could be quickly generated.

During accelerated corrosion testing, a white solid was observed to cover the pit cavity with evidence of some red/brown coloured Cu_2_O deposits. This is in agreement with previous reports on copper pitting mechanisms [25,26,27], where the pit interior is covered with solid CuCl thus hindering the formation of a protective Cu_2_O layer at the metal interface and also the low solubility of CuCl maintains a low activity of copper ions, thereby facilitating anodic dissolution. The formation of CuCl is often attributed to local conditions that restrict diffusion, for example a Cu_2_O layer. Lucey reported that pitting required the formation of a Cu_2_O layer that is sufficiently porous to allow the restricted diffusion of CuCl_2_^−^ through it and showed that the Cu_2_O behaves as an electrically conductive layer, with the upper surface acting as a cathode and the lower surface as an anode [27]. Thus, the CuCl trapped under the Cu_2_O layer may be anodically oxidised to cupric on the lower surface of the Cu_2_O layer and the resultant cupric ions can further attack the metallic copper alloy to reform cuprous ions. However, due to the restricted diffusion through the porous Cu_2_O layer and the block of accumulated CuCl solid in the cavity, the corrosion rate will decrease to a very low level in the long run as observed from current response with corrosion times, as shown in Figure 8.

**Figure 9:**
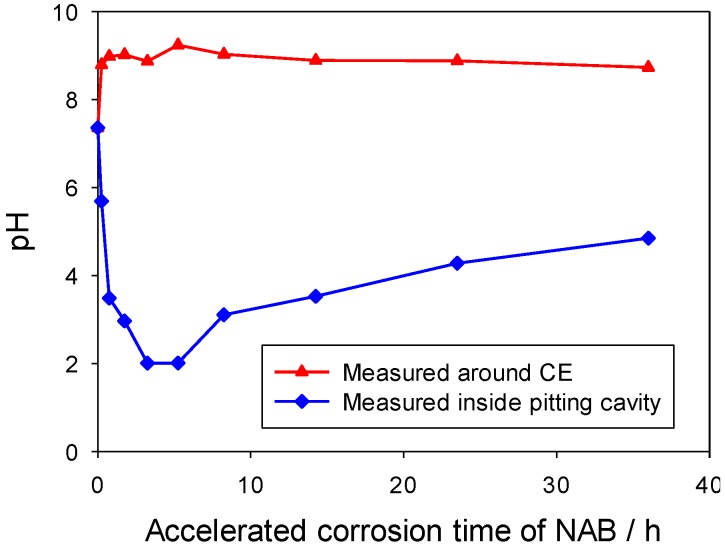
pH changes at pitting cavity site and around counter electrode with accelerated corrosion time at applied potential of +0.730 V for NAB pencil electrode in 3.5% NaCl solution.

The solution pH around counter the electrode became alkaline due to the cathodic oxygen reduction reaction, while the solution at the accelerated corrosion cavity became acidic due to hydrolysis of corrosion products, see Figure 9. It is noteworthy that the pH measured at the NAB corrosion site decreased to pH 2 during the initial stage of artificial pitting corrosion, and then rose slightly to pH 5. The lower pH levels in the NAB pitting cavity in comparison with the carbon steels may have resulted from the hydrolysis of other metal cations, such as the aluminium species.


Al + 4Cl^−^ → AlCl_4_^−^ + 3e^−^ (9)

AlCl_4_^−^+ 3H_2_O → Al(OH)_3_ + 3H^+^ + 4Cl^−^ (10)

**Figure 10:**
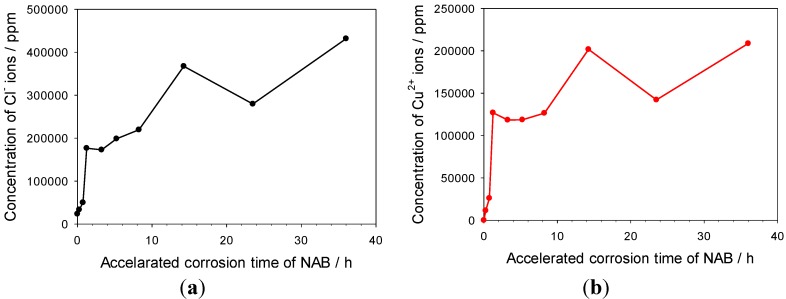
Concentration profiles of (**a**) chloride and (**b**) cupric ions as a function of accelerated corrosion time for NAB in 3.5% NaCl solution.

Figure 10 shows the chloride and cupric ion concentrations as a function of time. Due to the entrapment effect of the porous Cu_2_O layer, the cupric ions, in addition to the chloride, were able to accumulate and concentrate under this layer. After 14 h, the chloride and cupric ion concentrations reached around 432,000 ppm (*ca.* 12 M in terms of NaCl) and 208,000 ppm (*ca.* 3.3 M in terms of CuCl_2_^−^), respectively. In addition, metal ions for other alloy components including nickel, manganese, iron and aluminium found within NAB were also detected in the accelerated corrosion solution, and their concentrations reached the maximum within the initial 5 h. The maximum concentration of nickel ions detected in NAB accelerated corrosion solution is about 600 ppm, and the concentrations for manganese ions, ferrous ions and aluminium ions lower than 100 ppm.

## 4. Conclusions

A capillary electrophoresis methodology has been established with contactless conductivity detection for direct simultaneous determination of chloride ions and seven metal ions of the most commonly used alloy elements in a single run. The direct and simultaneous detection of all seven metal ions and chloride ions was achieved with a buffer solution of 10 mM PDCA and 0.5 mM CTAH at pH 4 using a pre-column complexation method and with the analysis performed at a temperature of 20 °C with an applied voltage of −15 kV. The detection limits obtained in this study for the metal cations and chloride ions were 100 ppb and 10 ppb, respectively. The established methodology has been demonstrated to be a versatile technique capable of speciation and quantifying the corrosive species generated within microenvironment chemistries found in artificial pit (a pencil electrode) and crevice corrosion geometries for carbon steels and nickel-aluminium bronze. Ultimately, knowledge of the metal ions, as well as chloride ion, concentration *vs*. time profiles during the corrosion processes will assist in the identification of the key corrosion analyte targets for critical aerospace, marine and microenvironments for structural health monitoring. In light of our results demonstrating contactless conductivity as a preferred detection methodology for capillary electrophoresis systems, we envisage a time when micro-CE systems with CCD could be feasibly used in autonomous, *in-situ* structural health monitoring.
